# 2-(5-Cyclo­hexyl-3-methyl­sulfanyl-1-benzofuran-2-yl)acetic acid

**DOI:** 10.1107/S1600536811026298

**Published:** 2011-07-09

**Authors:** Pil Ja Seo, Hong Dae Choi, Byeng Wha Son, Uk Lee

**Affiliations:** a Department of Chemistry, Dongeui University, San 24 Kaya-dong Busanjin-gu, Busan 614-714, Republic of Korea; b Department of Chemistry, Pukyong National University, 599-1 Daeyeon 3-dong, Nam-gu, Busan 608-737, Republic of Korea

## Abstract

In the title compound, C_17_H_20_O_3_S, the cyclo­hexyl ring adopts a chair conformation. In the crystal, the carboxyl groups are involved in inter­molecular O—H⋯O hydrogen bonds, which link the mol­ecules into centrosymmetric dimers. These dimers are further stabilized by weak inter­molecular C—H⋯O hydrogen bonds. In addition, the crystal structure also exhibits aromatic π–π inter­actions between the furan rings of adjacent mol­ecules [centroid–centroid distance = 3.505 (2) Å, inter­planar distance = 3.385 (2) Å and slippage = 0.909 (2) Å], and inter­molecular C—H⋯π inter­actions.

## Related literature

For the pharmacological activity of benzofuran compounds, see: Aslam *et al.* (2009 ▶); Galal *et al.* (2009 ▶); Khan *et al.* (2005 ▶). For natural products with benzofuran rings, see: Akgul & Anil (2003 ▶); Soekamto *et al.* (2003 ▶). For structural studies of related 2-(5-alkyl-3-methyl­sulfanyl-1-benzofuran-2-yl) acetic acid derivatives, see: Choi *et al.* (2009**a* ▶,b* ▶); Seo *et al.* (2007 ▶).
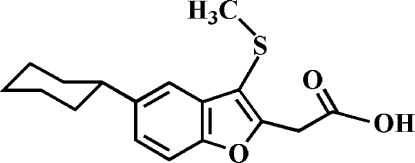


## Experimental

### 

#### Crystal data

C_17_H_20_O_3_S
*M* *_r_* = 304.40
Triclinic, 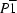

*a* = 7.3434 (2) Å
*b* = 9.0765 (3) Å
*c* = 11.6009 (4) Å
α = 86.086 (2)°
β = 86.083 (2)°
γ = 86.690 (2)°
*V* = 768.53 (4) Å^3^
*Z* = 2
Mo *K*α radiation
μ = 0.22 mm^−1^
*T* = 173 K
0.32 × 0.21 × 0.10 mm

#### Data collection

Bruker SMART APEXII CCD diffractometer
Absorption correction: multi-scan (*SADABS*; Bruker, 2009 ▶) *T*_min_ = 0.934, *T*_max_ = 0.978
14240 measured reflections
3864 independent reflections
3255 reflections with *I* > 2σ(*I*)
*R*_int_ = 0.030

#### Refinement

*R*[*F* ^2^ > 2σ(*F* ^2^)] = 0.038
*wR*(*F* ^2^) = 0.100
*S* = 1.05
3864 reflections
191 parameters
H-atom parameters constrained
Δρ_max_ = 0.30 e Å^−3^
Δρ_min_ = −0.26 e Å^−3^

### 

Data collection: *APEX2* (Bruker, 2009 ▶); cell refinement: *SAINT* (Bruker, 2009 ▶); data reduction: *SAINT*; program(s) used to solve structure: *SHELXS97* (Sheldrick, 2008 ▶); program(s) used to refine structure: *SHELXL97* (Sheldrick, 2008 ▶); molecular graphics: *ORTEP-3* (Farrugia, 1997 ▶) and *DIAMOND* (Brandenburg, 1998 ▶); software used to prepare material for publication: *SHELXL97*.

## Supplementary Material

Crystal structure: contains datablock(s) global, I. DOI: 10.1107/S1600536811026298/rk2282sup1.cif

Structure factors: contains datablock(s) I. DOI: 10.1107/S1600536811026298/rk2282Isup2.hkl

Supplementary material file. DOI: 10.1107/S1600536811026298/rk2282Isup3.cml

Additional supplementary materials:  crystallographic information; 3D view; checkCIF report

## Figures and Tables

**Table 1:** Hydrogen-bond geometry (Å, °) *Cg*1 and *Cg*2 are the centroids of the C1/C2/C7/O1/C8 furan ring and C2–C7 benzene ring, respectively.

*D*—H⋯*A*	*D*—H	H⋯*A*	*D*⋯*A*	*D*—H⋯*A*
C6—H6⋯O3^i^	0.95	2.59	3.4777 (17)	157
O3—H3*O*⋯O2^ii^	0.84	1.80	2.6347 (15)	176
C13—H13*A*⋯*Cg*1^iii^	0.99	2.82	3.581 (2)	134
C14—H14*B*⋯*Cg*2^iii^	0.99	2.85	3.678 (2)	147
C15—H15*A*⋯*Cg*2^iv^	0.99	2.67	3.501 (2)	142

Symmetry codes: (i) 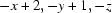; (ii) 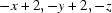; (iii) 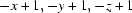; (iv) 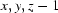.

**Fig. 1.**
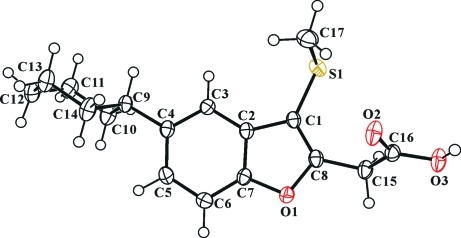
The molecular structure of the title compound with the atom numbering scheme. Displacement ellipsoids are drawn at the 50% probability level. H atoms are presented as a small spheres of arbitrary radius.

**Fig. 2.**
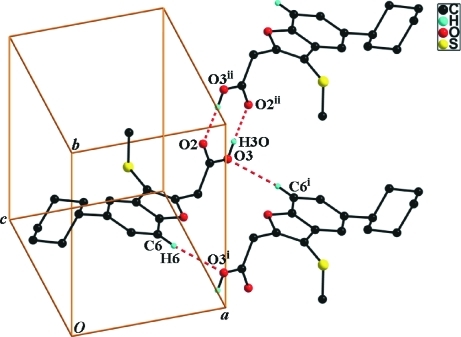
A view of the O—H···O and C—H···O inetractions (dotted lines) in the crystal structure of the title compound. Symmetry codes: (i) -x+2, -y+1, -z; (ii) -x+2, -y+2, -z.

**Fig. 3.**
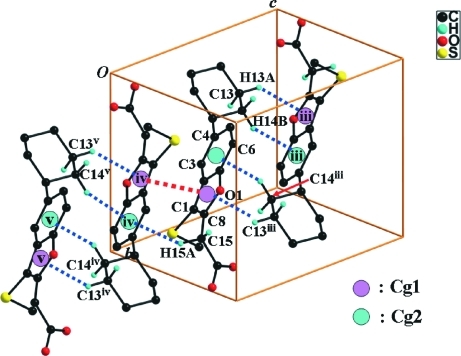
A view of the π–π and C—H···π inetractions (dotted lines) in the crystal structure of the title compound. Symmetry codes: (iii) -x+1, -y+1, -z+1; (iv) -x+1, -y+1, -z; (v) x, y, z-1.

## supplementary crystallographic information

### Comment

Recently, many compounds containing a benzofuran moiety have drawn much attention in view of their valuable pharmacological properties such as antibacterial and antifungal, antitumor and antiviral, and antimicrobial activities (Aslam *et al.*, 2009, Galal *et al.*, 2009, Khan *et al.*, 2005). These benzofuran derivatives occur in a wide range of natural products (Akgul & Anil, 2003; Soekamto *et al.*, 2003). As a part of our ongoing study of the substituent effect on the solid state structures of 2-(5-alkyl-3-methylsulfanyl-1-benzofuran-2-yl)acetic acid analogues (Choi *et al.*, 2009**a*,b*, Seo *et al.*, 2007), we report herein the crystal structure of the title compound.

In the title molecule, Fig. 1, the benzofuran unit is essentially planar, with a mean deviation of 0.006 (1)Å from the least-squares plane defined by the nine constituent atoms. The cyclohexyl ring has the *chair*-conformation. In the crystal structure, the carboxyl groups are involved in intermolecular O—H···O hydrogen bonds (Fig. 2 & Table 1), which link the molecules into centrosymmetric dimers. These dimers are further stabilized by weak intermolecular C—H···O hydrogen bonds between a benzene H atom and the O atom of the hydroxy group (Fig. 2 & Table 1; C6—H6···O3^i^). The crystal packing, Fig. 3, also exhibits aromatic π–π interactions between the furan rings of the adjacent molecules, with a Cg1···Cg1^iv^ distance of 3.505 (2)Å and an interplanar distance of 3.385 (2)Å resulting in a slippage of 0.909 (2)Å (Cg1 is the centroid of C1/C2/C7/O1/C8 furan ring). Additionally, the crystal packing (Fig. 3) shows intermolecular C—H···π interactions; the first one between a cyclohexyl H atom and the furan ring (Table 1; C13—H13A···Cg1^iii^), the second one between a cyclohexyl H atom and the benzene ring (Table 1; C14—H14B···Cg2^iii^), and the third one between an H atom of the benzylic methylene group and the benzene ring (Table 1; C15—H15A···Cg2^iv^, Cg2 is the centroid of the C2–C7 benzene ring). Symmetry codes as in the Table 1 and Fig. 2 and Fig. 3.

### Experimental

Ethyl 2-(5-cyclohexyl-3-methylsulfanyl-1-benzofuran-2-yl)acetate (398 mg, 1.2 mmol) was added to a solution of potassium hydroxide (337 mg, mmol) in water (10 ml) and methanol (10 ml), and the mixture was refluxed for 5h, then cooled. Water was added, and the solution was extracted with dichloromethane. The aqueous layer was acidified to pH = 1 with concentrated hydrochloric acid and then extracted with chloroform, dried over magnesium sulfate, filtered and concentrated at reduced pressure. The residue was purified by column chromatography (ethyl acetate) to afford the title compound as a colourless solid [yield 83%, m.p. 423–424 K; *R*_f_ = 0.45 (ethyl acetate)]. Single crystals suitable for X-ray diffraction were prepared by evaporation of a solution of the title compound in diisopropyl ether at room temperature.

### Refinement

All  H atoms were  positioned geometrically and refined using a riding model, with O—H = 0.84Å, and C—H = 0.95Å for aryl, 1.00Å for methine, 0.99Å for methylene and 0.98Å for methyl H atoms, respectively. *U*_iso_(H)  = 1.5*U*_eq_(O), and 1.2*U*_eq_(C) for aryl, methine, and methylene and 1.5*U*_eq_(C) for methyl H atoms.

### Crystal data

**Table:**

C_17_H_20_O_3_S	*Z* = 2
*M**_r_* = 304.40	*F*(000) = 324
Triclinic, *P*1	*D*_x_ = 1.315 Mg m^−^^3^
Hall symbol: -P 1	Melting point = 423–424 K
*a* = 7.3434 (2) Å	Mo *K*α radiation, λ = 0.71073 Å
*b* = 9.0765 (3) Å	Cell parameters from 6244 reflections
*c* = 11.6009 (4) Å	θ = 2.3–28.4°
α = 86.086 (2)°	µ = 0.22 mm^−^^1^
β = 86.083 (2)°	*T* = 173 K
γ = 86.690 (2)°	Block, colourless
*V* = 768.53 (4) Å^3^	0.32 × 0.21 × 0.10 mm

### Data collection

**Table:**

Bruker SMART APEXII CCD diffractometer	3864 independent reflections
Radiation source: rotating anode	3255 reflections with *I* > 2σ(*I*)
graphite multilayer	*R*_int_ = 0.030
Detector resolution: 10.0 pixels mm^-1^	θ_max_ = 28.6°, θ_min_ = 1.8°
φ and ω scans	*h* = −9→9
Absorption correction: multi-scan (*SADABS*; Bruker, 2009)	*k* = −9→12
*T*_min_ = 0.934, *T*_max_ = 0.978	*l* = −15→15
14240 measured reflections	

### Refinement

**Table:**

Refinement on *F*^2^	Primary atom site location: structure-invariant direct methods
Least-squares matrix: full	Secondary atom site location: difference Fourier map
*R*[*F*^2^ > 2σ(*F*^2^)] = 0.038	Hydrogen site location: difference Fourier map
*wR*(*F*^2^) = 0.100	H-atom parameters constrained
*S* = 1.05	*w* = 1/[σ^2^(*F*_o_^2^) + (0.0444*P*)^2^ + 0.2671*P*] where *P* = (*F*_o_^2^ + 2*F*_c_^2^)/3
3864 reflections	(Δ/σ)_max_ = 0.001
191 parameters	Δρ_max_ = 0.30 e Å^−^^3^
0 restraints	Δρ_min_ = −0.26 e Å^−^^3^

### Special details

**Table:**

Geometry. All s.u.'s (except the s.u. in the dihedral angle between two l.s. planes) are estimated using the full covariance matrix. The cell s.u.'s are taken into account individually in the estimation of s.u.'s in distances, angles and torsion angles; correlations between s.u.'s in cell parameters are only used when they are defined by crystal symmetry. An approximate (isotropic) treatment of cell s.u.'s is used for estimating s.u.'s involving l.s. planes.
Refinement. Refinement of *F*^2^ against ALL reflections. The weighted *R*-factor w*R* and goodness of fit *S* are based on *F*^2^, conventional *R*-factors *R* are based on *F*, with *F* set to zero for negative *F*^2^. The threshold expression of *F*^2^ > 2σ(*F*^2^) is used only for calculating *R*-factors(gt) etc. and is not relevant to the choice of reflections for refinement. *R*-factors based on *F*^2^ are statistically about twice as large as those based on *F*, and *R*-factors based on ALL data will be even larger.

### Fractional atomic coordinates and isotropic or equivalent isotropic displacement parameters (Å^2^)

**Table:**

	*x*	*y*	*z*	*U*_iso_*/*U*_eq_	
S1	0.39556 (5)	0.81571 (4)	0.07230 (3)	0.02882 (11)	
O1	0.75966 (12)	0.47823 (10)	0.08350 (8)	0.0242 (2)	
O2	0.88107 (14)	0.86762 (12)	0.06964 (8)	0.0308 (2)	
O3	0.96423 (14)	0.89156 (11)	−0.11894 (8)	0.0304 (2)	
H3O	1.0120	0.9672	−0.0995	0.046*	
C1	0.52945 (18)	0.65343 (14)	0.09979 (11)	0.0211 (3)	
C2	0.49420 (17)	0.53545 (14)	0.18707 (11)	0.0201 (3)	
C3	0.35767 (17)	0.50941 (14)	0.27432 (11)	0.0209 (3)	
H3	0.2577	0.5794	0.2852	0.025*	
C4	0.36936 (18)	0.37992 (14)	0.34536 (11)	0.0218 (3)	
C5	0.51779 (19)	0.27762 (15)	0.32655 (12)	0.0267 (3)	
H5	0.5243	0.1889	0.3749	0.032*	
C6	0.65490 (19)	0.30100 (15)	0.24036 (12)	0.0271 (3)	
H6	0.7544	0.2309	0.2283	0.032*	
C7	0.63899 (18)	0.43164 (14)	0.17300 (11)	0.0220 (3)	
C8	0.68847 (18)	0.61388 (14)	0.04136 (11)	0.0222 (3)	
C9	0.22459 (18)	0.35080 (15)	0.44187 (11)	0.0226 (3)	
H9	0.1408	0.4416	0.4441	0.027*	
C10	0.1076 (2)	0.22267 (17)	0.42058 (13)	0.0308 (3)	
H10A	0.1860	0.1305	0.4167	0.037*	
H10B	0.0519	0.2423	0.3453	0.037*	
C11	−0.0433 (2)	0.20246 (17)	0.51702 (13)	0.0329 (3)	
H11A	−0.1295	0.2905	0.5152	0.039*	
H11B	−0.1123	0.1154	0.5035	0.039*	
C12	0.0348 (2)	0.18098 (17)	0.63539 (13)	0.0339 (3)	
H12A	−0.0666	0.1757	0.6960	0.041*	
H12B	0.1085	0.0862	0.6406	0.041*	
C13	0.1536 (2)	0.30655 (19)	0.65673 (13)	0.0330 (3)	
H13A	0.2099	0.2854	0.7316	0.040*	
H13B	0.0765	0.3993	0.6617	0.040*	
C14	0.3035 (2)	0.32710 (19)	0.56051 (12)	0.0331 (3)	
H14A	0.3889	0.2386	0.5614	0.040*	
H14B	0.3734	0.4135	0.5746	0.040*	
C15	0.79618 (19)	0.68726 (15)	−0.05668 (12)	0.0250 (3)	
H15A	0.7147	0.7137	−0.1203	0.030*	
H15B	0.8927	0.6157	−0.0855	0.030*	
C16	0.88468 (17)	0.82431 (14)	−0.02779 (11)	0.0221 (3)	
C17	0.4488 (2)	0.91892 (17)	0.19209 (14)	0.0352 (3)	
H17A	0.4242	0.8600	0.2649	0.053*	
H17B	0.3729	1.0113	0.1924	0.053*	
H17C	0.5781	0.9414	0.1840	0.053*	

### Atomic displacement parameters (Å^2^)

**Table:**

	*U*^11^	*U*^22^	*U*^33^	*U*^12^	*U*^13^	*U*^23^
S1	0.0332 (2)	0.02291 (18)	0.0301 (2)	0.00036 (13)	−0.00535 (14)	0.00250 (13)
O1	0.0233 (5)	0.0225 (5)	0.0260 (5)	−0.0032 (4)	0.0036 (4)	0.0003 (4)
O2	0.0374 (6)	0.0353 (6)	0.0211 (5)	−0.0181 (4)	0.0030 (4)	−0.0037 (4)
O3	0.0382 (6)	0.0302 (5)	0.0235 (5)	−0.0169 (4)	0.0045 (4)	−0.0020 (4)
C1	0.0225 (6)	0.0191 (6)	0.0223 (6)	−0.0060 (5)	−0.0021 (5)	−0.0004 (5)
C2	0.0222 (6)	0.0191 (6)	0.0197 (6)	−0.0061 (5)	−0.0025 (5)	−0.0013 (5)
C3	0.0204 (6)	0.0210 (6)	0.0215 (6)	−0.0034 (5)	−0.0011 (5)	−0.0021 (5)
C4	0.0236 (6)	0.0223 (6)	0.0201 (6)	−0.0067 (5)	−0.0013 (5)	−0.0012 (5)
C5	0.0305 (7)	0.0226 (6)	0.0262 (7)	−0.0026 (5)	−0.0007 (6)	0.0036 (5)
C6	0.0264 (7)	0.0233 (7)	0.0304 (7)	0.0017 (5)	0.0007 (6)	0.0006 (6)
C7	0.0216 (6)	0.0230 (6)	0.0215 (6)	−0.0057 (5)	0.0008 (5)	−0.0011 (5)
C8	0.0257 (6)	0.0200 (6)	0.0215 (6)	−0.0069 (5)	−0.0019 (5)	−0.0012 (5)
C9	0.0237 (6)	0.0233 (6)	0.0208 (6)	−0.0061 (5)	0.0000 (5)	0.0008 (5)
C10	0.0330 (8)	0.0339 (8)	0.0270 (7)	−0.0150 (6)	0.0009 (6)	−0.0044 (6)
C11	0.0315 (8)	0.0336 (8)	0.0346 (8)	−0.0160 (6)	0.0034 (6)	−0.0037 (6)
C12	0.0361 (8)	0.0329 (8)	0.0310 (8)	−0.0076 (6)	0.0071 (6)	0.0055 (6)
C13	0.0322 (8)	0.0452 (9)	0.0217 (7)	−0.0085 (6)	−0.0013 (6)	0.0012 (6)
C14	0.0285 (7)	0.0485 (9)	0.0233 (7)	−0.0132 (6)	−0.0018 (6)	0.0002 (6)
C15	0.0288 (7)	0.0239 (6)	0.0227 (7)	−0.0088 (5)	0.0024 (5)	−0.0023 (5)
C16	0.0196 (6)	0.0233 (6)	0.0233 (6)	−0.0038 (5)	0.0000 (5)	0.0008 (5)
C17	0.0402 (9)	0.0269 (7)	0.0388 (9)	0.0018 (6)	−0.0023 (7)	−0.0063 (6)

### Geometric parameters (Å, °)

**Table:**

S1—C1	1.7463 (13)	C9—H9	1.0000
S1—C17	1.8044 (16)	C10—C11	1.530 (2)
O1—C7	1.3783 (15)	C10—H10A	0.9900
O1—C8	1.3808 (16)	C10—H10B	0.9900
O2—C16	1.2200 (16)	C11—C12	1.520 (2)
O3—C16	1.3041 (16)	C11—H11A	0.9900
O3—H3O	0.8400	C11—H11B	0.9900
C1—C8	1.3523 (19)	C12—C13	1.518 (2)
C1—C2	1.4446 (17)	C12—H12A	0.9900
C2—C7	1.3880 (18)	C12—H12B	0.9900
C2—C3	1.3934 (17)	C13—C14	1.523 (2)
C3—C4	1.3906 (18)	C13—H13A	0.9900
C3—H3	0.9500	C13—H13B	0.9900
C4—C5	1.4058 (19)	C14—H14A	0.9900
C4—C9	1.5109 (18)	C14—H14B	0.9900
C5—C6	1.3844 (19)	C15—C16	1.5040 (18)
C5—H5	0.9500	C15—H15A	0.9900
C6—C7	1.3786 (19)	C15—H15B	0.9900
C6—H6	0.9500	C17—H17A	0.9800
C8—C15	1.4823 (18)	C17—H17B	0.9800
C9—C14	1.5268 (19)	C17—H17C	0.9800
C9—C10	1.5280 (19)		
			
C1—S1—C17	100.05 (7)	C12—C11—C10	111.44 (12)
C7—O1—C8	105.90 (10)	C12—C11—H11A	109.3
C16—O3—H3O	109.5	C10—C11—H11A	109.3
C8—C1—C2	106.46 (11)	C12—C11—H11B	109.3
C8—C1—S1	125.80 (10)	C10—C11—H11B	109.3
C2—C1—S1	127.74 (10)	H11A—C11—H11B	108.0
C7—C2—C3	119.29 (12)	C13—C12—C11	111.38 (12)
C7—C2—C1	105.62 (11)	C13—C12—H12A	109.4
C3—C2—C1	135.08 (12)	C11—C12—H12A	109.4
C4—C3—C2	119.28 (12)	C13—C12—H12B	109.4
C4—C3—H3	120.4	C11—C12—H12B	109.4
C2—C3—H3	120.4	H12A—C12—H12B	108.0
C3—C4—C5	119.13 (12)	C12—C13—C14	111.49 (13)
C3—C4—C9	120.14 (12)	C12—C13—H13A	109.3
C5—C4—C9	120.73 (12)	C14—C13—H13A	109.3
C6—C5—C4	122.61 (13)	C12—C13—H13B	109.3
C6—C5—H5	118.7	C14—C13—H13B	109.3
C4—C5—H5	118.7	H13A—C13—H13B	108.0
C7—C6—C5	116.27 (13)	C13—C14—C9	111.51 (12)
C7—C6—H6	121.9	C13—C14—H14A	109.3
C5—C6—H6	121.9	C9—C14—H14A	109.3
O1—C7—C6	126.17 (12)	C13—C14—H14B	109.3
O1—C7—C2	110.43 (11)	C9—C14—H14B	109.3
C6—C7—C2	123.40 (12)	H14A—C14—H14B	108.0
C1—C8—O1	111.59 (11)	C8—C15—C16	114.63 (11)
C1—C8—C15	132.48 (13)	C8—C15—H15A	108.6
O1—C8—C15	115.93 (11)	C16—C15—H15A	108.6
C4—C9—C14	112.67 (11)	C8—C15—H15B	108.6
C4—C9—C10	112.85 (11)	C16—C15—H15B	108.6
C14—C9—C10	110.37 (12)	H15A—C15—H15B	107.6
C4—C9—H9	106.8	O2—C16—O3	124.13 (12)
C14—C9—H9	106.8	O2—C16—C15	123.70 (12)
C10—C9—H9	106.8	O3—C16—C15	112.17 (11)
C9—C10—C11	111.07 (12)	S1—C17—H17A	109.5
C9—C10—H10A	109.4	S1—C17—H17B	109.5
C11—C10—H10A	109.4	H17A—C17—H17B	109.5
C9—C10—H10B	109.4	S1—C17—H17C	109.5
C11—C10—H10B	109.4	H17A—C17—H17C	109.5
H10A—C10—H10B	108.0	H17B—C17—H17C	109.5
			
C17—S1—C1—C8	−104.94 (13)	S1—C1—C8—O1	−179.32 (9)
C17—S1—C1—C2	75.49 (13)	C2—C1—C8—C15	−179.89 (13)
C8—C1—C2—C7	−0.22 (14)	S1—C1—C8—C15	0.5 (2)
S1—C1—C2—C7	179.42 (10)	C7—O1—C8—C1	−0.29 (14)
C8—C1—C2—C3	179.08 (14)	C7—O1—C8—C15	179.88 (11)
S1—C1—C2—C3	−1.3 (2)	C3—C4—C9—C14	−122.24 (14)
C7—C2—C3—C4	−0.06 (18)	C5—C4—C9—C14	57.33 (17)
C1—C2—C3—C4	−179.28 (13)	C3—C4—C9—C10	111.93 (14)
C2—C3—C4—C5	−0.68 (19)	C5—C4—C9—C10	−68.49 (16)
C2—C3—C4—C9	178.90 (11)	C4—C9—C10—C11	−176.96 (12)
C3—C4—C5—C6	0.6 (2)	C14—C9—C10—C11	55.98 (16)
C9—C4—C5—C6	−178.95 (13)	C9—C10—C11—C12	−55.76 (17)
C4—C5—C6—C7	0.2 (2)	C10—C11—C12—C13	54.84 (17)
C8—O1—C7—C6	179.81 (13)	C11—C12—C13—C14	−54.64 (18)
C8—O1—C7—C2	0.14 (14)	C12—C13—C14—C9	55.54 (18)
C5—C6—C7—O1	179.39 (12)	C4—C9—C14—C13	176.83 (12)
C5—C6—C7—C2	−1.0 (2)	C10—C9—C14—C13	−56.01 (17)
C3—C2—C7—O1	−179.39 (11)	C1—C8—C15—C16	69.12 (19)
C1—C2—C7—O1	0.04 (14)	O1—C8—C15—C16	−111.10 (13)
C3—C2—C7—C6	0.9 (2)	C8—C15—C16—O2	5.1 (2)
C1—C2—C7—C6	−179.63 (13)	C8—C15—C16—O3	−174.44 (12)
C2—C1—C8—O1	0.32 (15)		

### Hydrogen-bond geometry (Å, °)

**Table:**

Cg1 and Cg2 are the centroids of the C1/C2/C7/O1/C8 furan ring and C2–C7 benzene ring, respectively.

**Table:**

*D*—H···*A*	*D*—H	H···*A*	*D*···*A*	*D*—H···*A*
C6—H6···O3^i^	0.95	2.59	3.4777 (17)	157
O3—H3O···O2^ii^	0.84	1.80	2.6347 (15)	176
C13—H13A···Cg1^iii^	0.99	2.82	3.581 (2)	134
C14—H14B···Cg2^iii^	0.99	2.85	3.678 (2)	147
C15—H15A···Cg2^iv^	0.99	2.67	3.501 (2)	142

Symmetry codes: (i) −*x*+2, −*y*+1, −*z*; (ii) −*x*+2, −*y*+2, −*z*; (iii) −*x*+1, −*y*+1, −*z*+1; (iv) *x*, *y*, *z*−1.
